# The role of fibroblast growth factor 8 in cartilage development and disease

**DOI:** 10.1111/jcmm.17174

**Published:** 2022-01-09

**Authors:** Haoran Chen, Yujia Cui, Demao Zhang, Jing Xie, Xuedong Zhou

**Affiliations:** ^1^ State Key Laboratory of Oral Diseases West China Hospital of Stomatology Sichuan University Chengdu China; ^2^ Department of Cariology and Endodontics West China Hospital of Stomatology Sichuan University Chengdu China

**Keywords:** cartilage, chondrocyte, FGF‐8, osteoarthritis, skeletal system

## Abstract

Fibroblast growth factor 8 (FGF‐8), also known as androgen‐induced growth factor (AIGF), is presumed to be a potent mitogenic cytokine that plays important roles in early embryonic development, brain formation and limb development. In the bone environment, FGF‐8 produced or received by chondrocyte precursor cells binds to fibroblast growth factor receptor (FGFR), causing different levels of activation of downstream signalling pathways, such as phospholipase C gamma (PLCγ)/Ca^2+^, RAS/mitogen‐activated protein kinase‐extracellular regulated protein kinases (RAS/MAPK‐MEK‐ERK), and Wnt‐β‐catenin‐Axin2 signalling, and ultimately controlling chondrocyte proliferation, differentiation, cell survival and migration. However, the molecular mechanism of FGF‐8 in normal or pathological cartilage remains unclear, and thus, FGF‐8 represents a novel exploratory target for studies of chondrocyte development and cartilage disease progression. In this review, studies assessing the relationship between FGF‐8 and chondrocytes that have been published in the past 5 years are systematically summarized to determine the probable mechanism and physiological effect of FGF‐8 on chondrocytes. Based on the existing research results, a therapeutic regimen targeting FGF‐8 is proposed to explore the possibility of treating chondrocyte‐related diseases.

## 1 INTRODUCTION

Healthy cartilage is the basis of joint development and physiological movement. The normal formation and maturation of cartilage require the coordination of a variety of signalling, including bone morphogenetic proteins (BMPs), transforming growth factor β (TGF‐β), parathyroid signalling, hedgehog (Hh) signalling, wingless‐type MMTV integration site (Wnt) signalling and fibroblast growth factor (FGF) signalling.^1^, ^2^, ^3^ Most of them have been well known, but FGF is not. Current reports have indicated that FGF signalling plays an indispensable role in maintaining joint health and functional homeostasis by regulating the cell behaviours of articular chondrocytes and peripheral synoviocytes and osteoblasts.^4^, ^5^ Moreover, abnormality of FGF signalling during development leads to cartilage atrophy.^6^ In the progress of cartilage diseases, FGF protein family is also recognized to play a potential role.^7^ However, at present, there is still lack of sufficient experimental or pathological evidence to explain the importance of FGF family in cartilage development and diseases.

Human FGF subfamily is a kind of cytokines that play important roles in cell growth, development, metabolism and tissue disease.^8^ There are 22 members in the FGF family (FGF‐1‐23 in human, lacking FGF‐15, because FGF‐15 is a mouse homologous gene of human FGF‐19^9^), which can be divided into two categories: paracrine and endocrine. According to the similarity and specificity of its protein structure, FGF family is divided into seven subfamilies, namely FGF‐1, FGF‐4, FGF‐7, FGF‐8, FGF‐9, FGF‐11 and FGF‐19 subfamilies. Among them, FGF‐1 subfamily (including FGF‐1 and FGF‐2), FGF‐4 subfamily (including FGF‐4, FGF‐5 and FGF‐6), FGF‐7 subfamily (including FGF‐3, FGF‐7, FGF‐10 and FGF‐22), FGF‐8 subfamily (including FGF‐8, FGF‐17 and FGF‐18), FGF‐9 subfamily (including FGF‐9, FGF‐16 and FGF‐20) and FGF‐11 subfamily (including FGF‐11–14) belong to paracrine class, while FGF‐19 subfamily (including FGF‐19, FGF‐21 and FGF‐23) belong to the endocrine category.^8^, ^9^, ^10^, ^11^ Current studies have shown that FGF members, such as FGF‐2, FGF‐9, FGF‐18 and FGF‐19, have been closely implicated into the physiology and pathology of cartilage and they can promote the development of cartilage and bone,^1^, ^9^, ^12^, ^13^, ^14^, ^15^, ^16^ but the specific role of FGF‐8 subfamily in the growth and development of cartilage and cartilage disease progression remains partially known. The FGF‐8 subfamily consists of three proteins: FGF‐8, FGF‐17 and FGF‐18.^9^, ^12^ Among them, FGF‐17 plays a vital role in brain development,^17^ while FGF‐18 and FGF‐8 play important roles in chondrogenesis and osteogenesis.^12^, ^18^, ^19^ Considering the limited understandings of FGF‐18 in cartilage formation and repair,^1^, ^8^ we can deduce the importance of FGF‐8 in the physiology and pathology of cartilage. Thus, in this review, we summarize the background of FGF‐8 and its receptors (FGFRs) in articular cartilage development, homeostasis and related cartilage diseases, discuss the current research of OA and cartilage injury based on FGF‐8, and emphasize the future challenges in this field.

**FIGURE 1:**
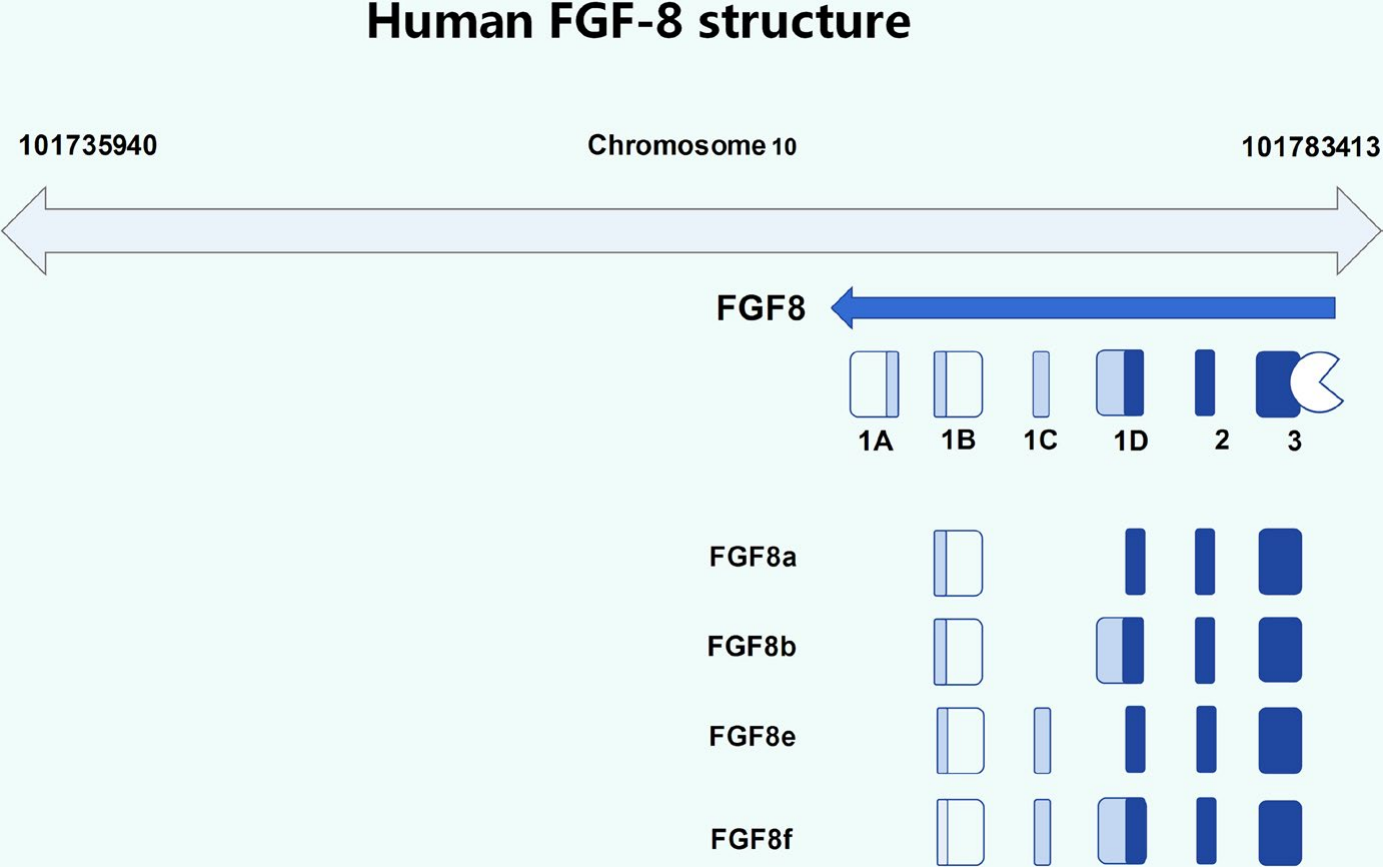
Structure of FGF‐8. (A) The FGF‐8 gene is a six‐part segment on chromosome 10, of which 1A, 1B, 1D and 3 are composed of two smaller segments. (B) FGF‐8a, FGF‐8b, FGF‐8e and FGF‐8f in humans are all encoded by FGF gene fragments 1B, 2 and 3, and the difference is in the composition of the 1C and 1D segments

**FIGURE 2:**
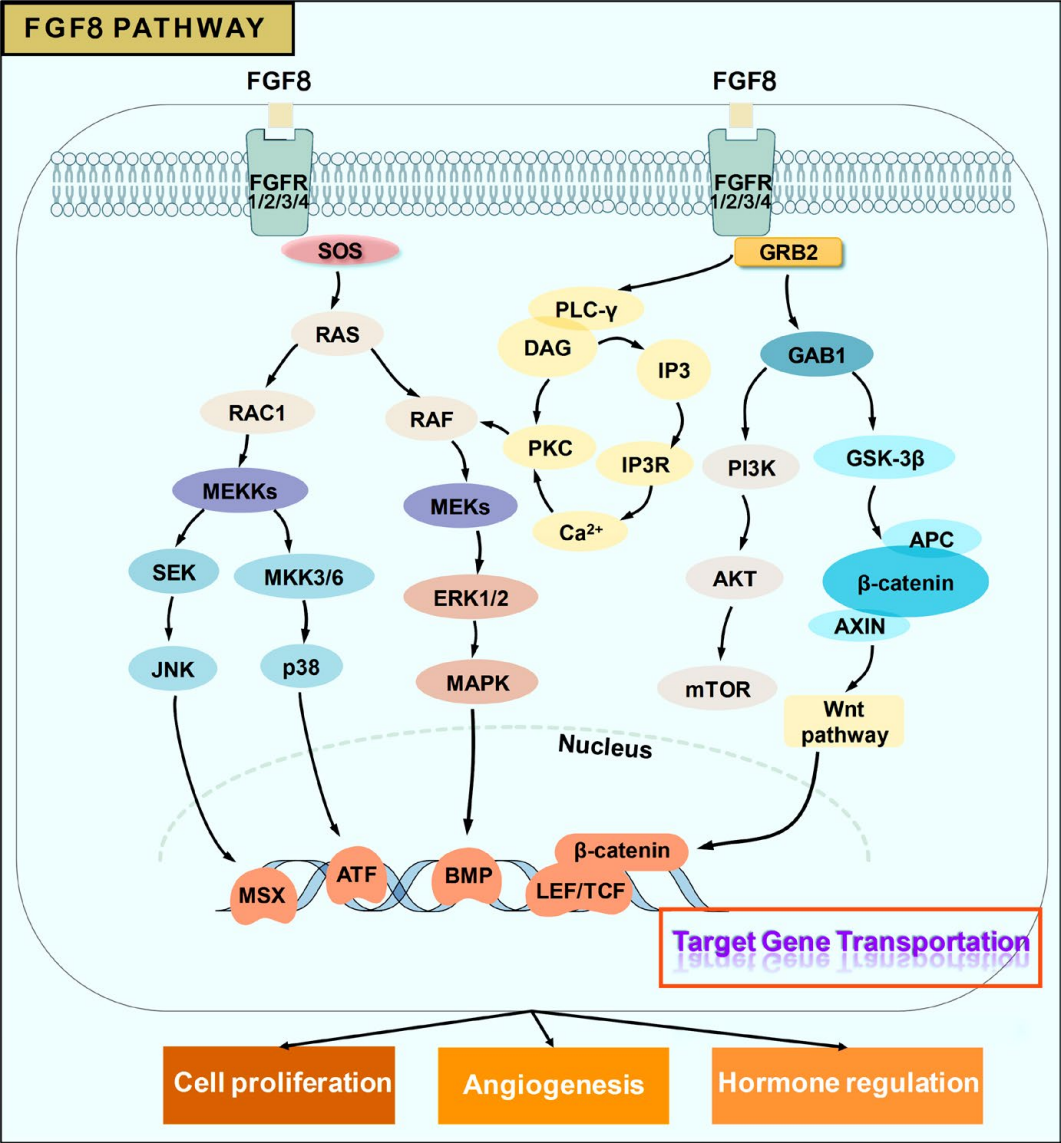
FGF‐8‐related signalling pathway. (A) The binding of FGF‐8 molecules to FGFR activates a series of signalling pathways, such as PI3K/AKT, PLCγ/Ca^2+^, RAS/MAPK, MEK‐ERK and Wnt‐β‐catenin‐Axin2. (B) The MAPK‐ERK‐MEK pathway and Wnt signalling pathway induced by FGF‐8 signalling exert both promoting and inhibitory effects and jointly coordinate angiogenesis, tissue development and hormone regulation in the body. (C) The Wnt signalling pathway and JNK signalling pathway also interact through the activation of FGF‐8 signalling pathway

The FGF‐8 protein was originally identified in a mouse model of androgen‐dependent breast cancer by Tanaka in 1992, and thus, FGF‐8 protein is also recognized as androgen‐induced growth factor (AIGF).^20^, ^21^ The FGF‐8 is involved in the activation of physiological cellular activities such as cell proliferation and differentiation, cell migration and the survival of early embryonic cells in the human body,^22^ and this partially determines its role in the morphological development of human embryos, limb maturation, differentiation and evolution of the nervous system, adolescent hormone regulation in postnatal development.^5^, ^23^, ^24^ The human FGF‐8 protein includes the isoforms FGF‐8A, FGF‐8B, FGF‐8E and FGF‐8F, among which FGF‐8B is considered to have the strongest ability to recognize and bind FGFR (Figure 1). FGF‐8, especially FGF‐8B, exerts different biological effects on humans by binding to different FGFR isoforms.^20^ FGF‐8 family members show a unique binding affinity for FGF receptors and tissue distribution patterns.^12^, ^22^, ^25^ In normal physiology, FGFR consists mainly of 4 members, FGFR1, FGFR2, FGFR3 and FGFR4, with corresponding subclasses of FGFR2 and FGFR3.^8^, ^26^ FGF‐8 mutual recognition and binding to FGFR results in varying degrees of activation of downstream signalling pathways, such as the RAS/MAPK, MEK‐ERK, Wnt‐β‐catenin and PLC‐γ/Ca^2+^ pathways, activating downstream factors such as MSX‐1, BMP4 and Wnt‐β‐catenin to promote corresponding physiological activities such as angiogenesis and hormonal regulation (Figure 2).^13^, ^20^, ^27^, ^28^, ^29^, ^30^ FGF‐8 can promote angiogenesis and there is angiogenesis in joint inflammation such as osteoarthritis, so we speculated that when FGF‐8, especially FGF‐8B, is overexpressed, it may potentially promote the occurrence and development of joint inflammation.^20^, ^31^, ^32^ All current researches indicate that in‐depth insights into the molecular mechanism of the FGF‐8 signalling pathway are urgently needed to provide a better understanding of FGF‐8 in human growth, inflammatory process and even the potential personalized therapy.

## 2 OVERVIEW OF FGF‐8 IN NORMAL CARTILAGE

### 2.1 The cartilage tissue

Cartilage tissue is composed of scattered low‐density single chondrocytes and abundant cartilage matrix, which contains no blood vessels, lymphoid tissue or nerves.^33^, ^34^ Cartilage is a supportive cellular connective tissue with a tough texture. According to the difference in cartilage matrix, cartilage is divided into three types: hyaline, elastic and fibrous. Among them, hyaline cartilage is widely studied because of its ability to secrete extracellular matrix (ECM).^35^ Accumulated evidence has shown that FGF signalling pathways play an important role in cartilage production, maturation and development of subchondral bone.^36^, ^37^ Among them, fibroblast growth factor 2 and 19 (FGF‐2 and FGF‐19) have been studied extensively, but FGF‐8 interacted with chondrocytes is not completely understood. Therefore, the molecular mechanism of FGF‐8 in cartilage tissue has been extensively investigated experimentally in recent years to determine the interaction between FGF‐8 and cartilage tissue.

### 2.2 The importance of FGF‐8 in cartilage physiology

**FIGURE 3:**
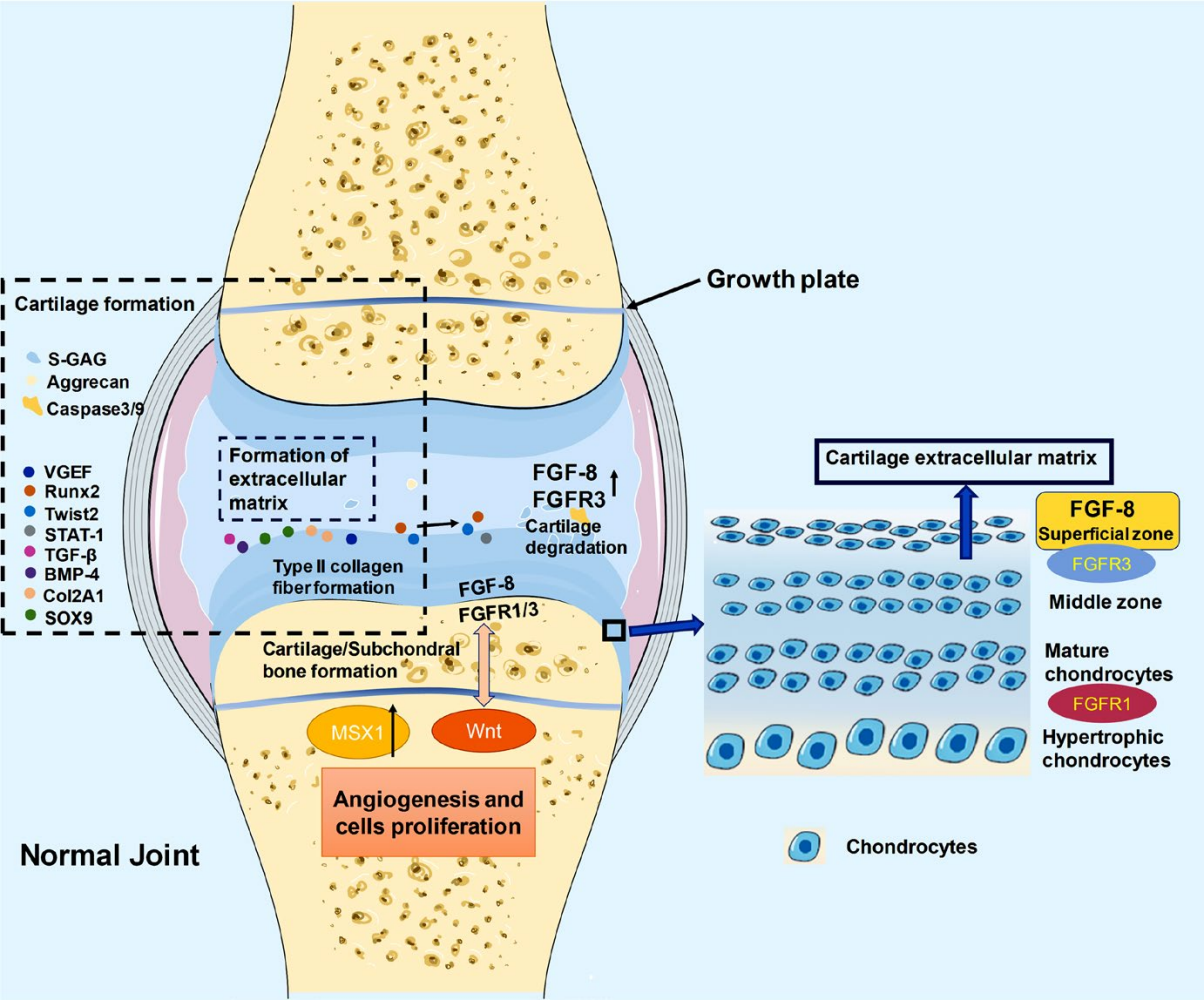
Role of FGF‐8 in normal cartilage. (A) In the initial stage of chondrogenesis, mesenchymal cells from the neural crest migrate towards the articular site through the actions of FGF‐8, SHH and FGFR1, and differentiate into chondrogenic precursors. Chondrocytes then undergo early cell proliferation through mechanisms mediated by FGF‐8, FGFR3 and FGFR1. (B) Four layers of chondrocytes have been identified, among which FGFR1 is mainly expressed in mature chondrocytes and hypertrophic chondrocytes, while FGFR3 is expressed at higher levels in surface chondrocytes with more active proliferation. (C) In the process of cartilage development, FGFR3 both promotes and inhibits cartilage formation. In the early stage of chondrogenesis, the interaction of FGFR3 and FGF‐8 induces the expression of SOX9, COL2A1 and other chondrocyte markers and promotes the proliferation and differentiation of chondrocytes and the construction of extracellular matrix. FGF‐8 and FGFR3 induce the expression of osteoblastic markers such as Runx2 and Twist2, degrade aggrecan, and inhibit the synthesis of extracellular matrix by chondrocytes. Moreover, FGF‐8 and FGFR3 promote the apoptosis of chondrocytes by activating caspase3/9. These processes result in a dynamic balance of cartilage production and degradation in normal mature articular cartilage

The roles of FGF‐8 and FGFR in the development of normal cartilage tissue are manifested in the interaction in the dynamic balance of catabolism and anabolism.^5^, ^37^, ^38^, ^39^, ^40^ In different stages of the growth and development of limbs and joints, different types of FGFRs successively bind to FGF‐8 to activate downstream signalling pathways with different intensities.^8^, ^41^ The complex biological effects of all signalling pathways are cumulative and manifest as the ultimate result of limb and joint development. At the same time, FGF‐8 and FGFR are maintained in long‐term dynamic balance in normal mature cartilage tissue to ensure the normal motor function of the joints and limbs of the body (Figure 3).^23^, ^42^, ^43^, ^44^, ^45^ In the early stages of joint and limb development, mesenchymal cells from the trunk and head neural crest (TNC and HNC), which are induced by FGF‐8 (especially FGF‐8B which has been shown to induce differentiation of mesenchymal cells into chondrocytes recently^46^), sonic hedgehog (SHH) and FGFR1, migrate and condense to form growth plates, showing a strong potential to differentiate into cartilage and bone..^10^, ^23^, ^47^, ^48^ FGF‐8 is involved in the process of cartilage generation, which is mediated by the interaction between FGF‐8 and FGFR, especially FGFR1 and FGFR3. The effect is determined to the difference in signalling pathway strength; FGFR1 mainly regulates catabolism, while FGFR3 mainly regulates anabolism.^5^, ^8^, ^49^, ^50^, ^51^ FGFR3 is expressed in proliferating chondrocytes. FGFR2 is expressed slightly later than FGFR3 and functions similarly to FGFR3, while FGFR1 is expressed at higher levels in hypertrophic chondrocytes.^41^ Recent studies have found that FGF‐8 can cooperate with BMP2 in inducing the growth and development of trunk and articular cartilage, firstly upregulating SOX9 gene and type II collagenase A1 (Col2A1) to promote chondrogenesis. And in embryos with abnormal function and mutation, FGF‐8 can rescue RNA transcription so that indirectly regulates cartilage and osteogenic differentiation and rescues craniofacial and articular defects.^30^, ^32^, ^52^, ^53^, ^54^ These two factors are considered markers of cartilage production, which promote chondrocyte differentiation and produce type II collagen fibres necessary for the formation of chondrocyte extracellular matrix.^10^, ^52^, ^55^, ^56^ FGF‐8 binds to FGFR3 and FGFR1, which play a role in early chondrocyte proliferation, and activates the MAPK, MEK‐ERK, JNK and PI3K/Akt pathways and corresponding downstream molecules such as BMP‐7, GP130, MSX‐1 and VGEF, to induce the proliferation of immature chondrocytes and facilitate a repair on cartilage.^49^, ^57^, ^58^, ^59^ Activation of these molecules increases the migration of chondrocytes and promotes the secretion of aggrecan and chondroitin sulphate, which increase the proliferation of chondrocytes and form positive feedback.^13^, ^28^, ^57^, ^60^, ^61^, ^62^ In addition, FGF‐8 also promotes chondrocyte differentiation by activating the Smad4 and BMP4‐TGF‐β signalling pathways at an early stage.^22^, ^28^, ^54^, ^63^, ^64^, ^65^ As the cartilage in the growth plate continues to proliferate and differentiate, the chondrocytes gradually form four layers. FGFR1 is expressed in the lowest layer of hypertrophic chondrocytes and in prehypertrophic chondrocytes to produce type 10 collagen fibres, while FGFR3 continues to be expressed in the proliferative first layer of chondrocytes.^4^, ^36^, ^41^, ^66^ In normal chondrocytes, FGF‐8 is not expressed at high levels, and the proliferation and hypertrophy of chondrocytes are well controlled,^67^ potentially due to the dynamic balance of proliferation and apoptosis between the upper proliferative layer and the lower hypertrophic layer of chondrocytes.^45^ When FGF‐8 interacts with an FGFR3 mutant, ectopic cartilage and osteophytes are generated, resulting in Kashin‐Beck disease or abnormal cartilage and bone.^68^, ^69^ Simultaneously, overexpression of FGF‐8 in combination with FGFR causes abnormal cartilage proliferation and promotes a cartilage fate in normal tissues rather than osteogenesis.^70^, ^71^ Based on these findings, FGF‐8 and FGFR should exert a negative regulatory effect on the proliferation and differentiation of chondrocytes in normal cartilage tissue. At the late stage of chondrocyte proliferation, FGF‐8‐bound FGFR3 activates P38/P53 through the MAPK‐ERK signalling pathway and upregulates STAT1 to inhibit chondrocyte proliferation, differentiation and extracellular matrix synthesis by inducing the rapid loss of proteoglycan ECM in chondrocytes through the inhibition of matrix synthesis and activation of proteolytic degradation.^30^, ^60^, ^72^, ^73^, ^74^, ^75^, ^76^ At the same time, Caspase‐3/9 are upregulated to induce apoptosis of chondrocytes.^7^, ^24^, ^77^ After SOCS3 was appeared to reduce the duration and amplitude of the MAPK pathway, FGFR3 reduced the inhibition of premature proliferation of chondrocytes.^72^, ^75^ Additionally, the FGF signalling also inhibits chondrocyte proliferation through the STAT1 pathway.^78^ These experimental results indicate that in normal cartilage, the interaction between FGF‐8 and FGFR3 could promote and inhibit the proliferation and differentiation of chondrocytes. Other studies have shown that the interrelationship between the Wnt signalling pathway and FGF signalling pathway plays an important role in chondrogenesis.^79^, ^80^ The FGF signalling pathway activates the Wnt/β‐catenin pathway in the chondrocyte, resulting in a loss of extracellular matrix, the expression of genes typical of mineralized tissues and changes in cell morphology.^7^, ^39^, ^43^, ^67^, ^79^, ^80^, ^81^, ^82^ These results suggest that the classical Wnt signalling pathway exerts a negative feedback effect on regulating the proliferation and differentiation of FGF‐8‐stimulated chondrocytes. Wnt‐mTOR and NF‐κβ also produce acid‐sensing ion channels 1A (ASIC1A) under acidic conditions to promote the apoptosis of chondrocytes.^49^, ^50^, ^83^, ^84^ The expression of β‐catenin and Wnt3A is detected when FGF‐8 is expressed in cartilage tissue, and these molecules have been indicated to induce the expression of osteorix, an ossifying factor. Runx2 and Twist2 are produced, thereby limiting cartilage differentiation.^37^, ^42^, ^54^, ^85^, ^86^, ^87^ However, the lack of osteorix and β‐catenin leads to abnormal chondrogenic differentiation, and FGF‐8 upregulates Wnt5a and Wnt9a.^23^, ^54^, ^88^, ^89^, ^90^ Overexpression of FGF‐8 increases cartilage formation and disrupts the normal cartilage to bone ratio, while a reduction in the expression of Wnt‐Axin2 genes partially inhibits bone loss due to excessive cartilage formation,^70^ suggesting that a balance of positive and negative effects exists between the Wnt signalling pathway and FGF‐8‐guided FGFR pathway. In conclusion, during the development of normal cartilage tissue, cartilage tissue maintains a dynamic balance of anabolism and catabolism, and the molecular mechanism is based on the binding of FGF‐8 to FGFR receptors (mainly FGFR1 and FGFR3) and the subsequent activation of downstream MAPK signalling. The mechanism underlying the interaction between the MEK‐ERK and PI3K‐Akt signalling pathways and the Wnt signalling pathway in chondrocytes is complex. Cartilage eventually differentiates, grows healthily and becomes stable, which is closely related to the cooperation between all the aforementioned signalling pathways. Finally, cartilage tissue avoids abnormal proliferation and differentiation and maintains the normal metabolic state in the joint through strict regulation in the body.

## 3 THE RELATIONSHIP BETWEEN FGF‐8 AND CHONDROCYTES IN CARTILAGE DISEASES

Chondrocytes undergo many processes in developing into mature cartilage tissue, such as cell proliferation, cell differentiation, maturation and hypertrophy, and cartilage stromal cell aggregation.^5^, ^66^ There is only a single low‐density chondrocyte and extracellular matrix in cartilage tissue. Pathological cartilage formation or the progression of chondrocyte diseases may be related to the abnormal state of chondrocytes and chondrocyte matrix. At present, the relationship between FGF‐8 and cartilage diseases has been lucubrated, aiming to provide potential guidance for the diagnosis and treatment of cartilage diseases in future.

### 3.1 Osteoarthritis (OA)

**FIGURE 4:**
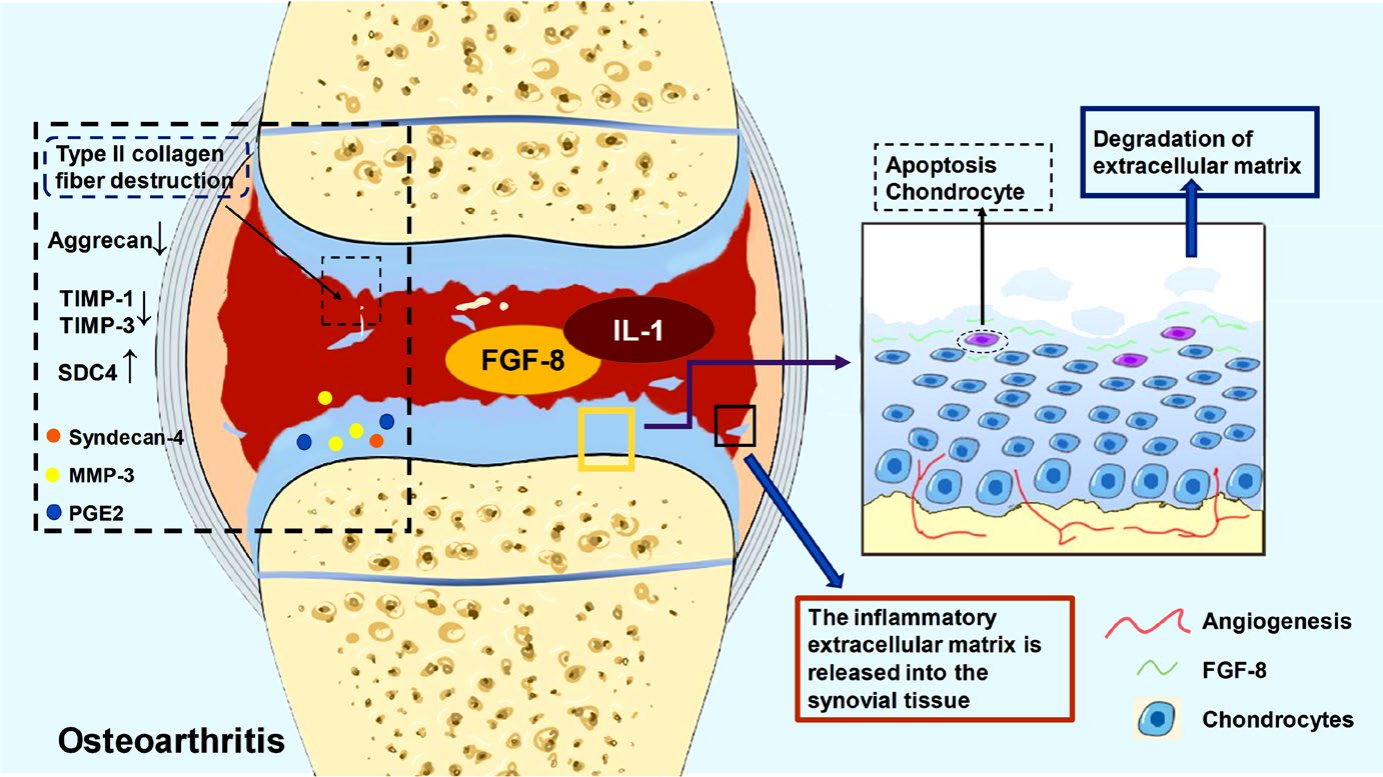
Role of FGF‐8 in osteoarthritis cartilage. (A) SDC4, which encodes Syndecan‐4, is overexpressed in cartilage from subjects with osteoarthritis, leading to the overexpression of its downstream putative factor MMP‐3. MMP‐3 degrades type II collagen and aggrecan in articular cartilage, leading to progressive cartilage damage. Meanwhile, tissue inhibitor of metalloproteinases 3 (TIMP‐3) and TIMP‐1, which limit MMP‐3, is downregulated, leading to chondrocyte damage. (B) Other factors, such as chondrocyte ageing, oxidative stress or inflammation, also inhibit the synthesis of glucosaminoglycans and type II collagen fibres and upregulate the expression of type I collagen, matrix metalloproteinase‐3 and proinflammatory cytokines through the activation of mitogen‐activated protein kinase (MAPK) and MAPK/ERK signalling pathways. (C) In the osteoarthritis model, the degradation of chondrocyte extracellular matrix and the release of the degraded matrix into synovial tissue, as well as the formation of inflammatory blood vessels, are the main causes of disease progression

The expression of FGF‐8 is low in mature cartilage tissue. However, in the rabbit osteoarthritis model constructed by Uchii et al. through meniscectomy in 2008, the level of FGF‐8 expression in synovial cells of cartilage was significantly increased, suggesting the potential pathological role of FGF‐8 in osteoarthritis. They also established an osteoarthritis model through an intracavitary injection of FGF‐8 or monoiodoacetate into the knee joint of rabbits. They found that injection of FGF‐8 into the knee joint of rats can cause ECM degradation, and its degradation can be inhibited by anti‐FGF‐8 antibody. In a monoiodoacetic acid‐induced osteoarthritis model, anti‐FGF‐8 antibodies reduced ECM release to the synovium, suggesting that the FGF‐8 may promote the degradation of ECM and damage cartilage structure in osteoarthritis (Figure 4).^91^, ^92^ The early stages of osteoarthritis are characterized by the loss of the extracellular matrix.^58^ In the normal articular cartilage matrix, the most important components are type II collagen fibres and aggrecans, and the synthesis and catabolism of type II collagen fibres and aggrecans maintain the dynamic balance and normal joint activities.^38^, ^65^ According to experimental results, syndecan‐4 gene (SDC4), which encodes syndecan‐4, is overexpressed in the cartilage of subjects with osteoarthritis, resulting in the overexpression of its downstream putative factor matrix metalloproteinase‐3 (MMP‐3).^85^ MMP‐3 causes progressive damage to the matrix of articular cartilage due to the degradation of type II collagen and aggrecan,^93^ with restrictions of the proliferation of chondromesenchymal cells mediated by endogenous inhibitors, tissue inhibitors of metalloproteinases 3 (TIMP‐3) and TIMP‐1 or factor CD34,^39^, ^94^, ^95^ thus resulting in damage to chondrocytes. Meanwhile, other factors, such as chondrocyte ageing, oxidative stress or inflammation, also inhibit the synthesis of glucosaminoglycans and type II collagen fibres and upregulate the expression of type I collagen, MMP‐3 and proinflammatory cytokines by activating the MAPK/ERK signalling pathways. Eventually, cartilage homeostasis shifts towards cartilage degradation.^58^, ^77^, ^84^, ^96^ When FGF‐8 is applied in combination with interleukin‐1 in vivo, the degradation of cartilage matrix in the inflammatory joints is enhanced because interleukin‐1 activates the JNK‐2 signalling pathway to induce aggrecan degradation on chondrocytes.^5^, ^61^, ^97^ In cell coculture experiments, FGF‐8‐induced chondrocytes to produce MMP‐3 and prostaglandin E2 (PGE2), two proteins that promote extracellular matrix degradation and increase the breakdown of type II collagen fibres.^92^, ^93^ Antibodies against FGF‐8, such as SU5402, were used to reduce the degradation of ECM and its release into synovial tissue.^5^, ^84^, ^91^ Based on these results, FGF‐8 may be involved in the aggravation of osteoarthritis, mainly in catabolism..^37^, ^68^ Recent studies have shown that the progression of osteoarthritis is related to the disorder of cellular metabolism, and FGF‐8 is involved in cartilage catabolic metabolism, which can be regarded as a potential therapeutic target for osteoarthritis.^84^ However, the specific molecular mechanisms, including the downstream signal transduction pathways activated by FGF‐8 in the progression of osteoarthritis and the specific binding of FGF receptor proteins, still require further investigation.

### 3.2 Kashin‐Beck disease

Kashin‐Beck disease is a comprehensive cartilage disease characterized by necrosis of the deep cells of the bone plate cartilage and articular cartilage, accompanied by secondary proliferation and changes in the repair of cartilage tissue, which is considered a unique type of osteoarthritis.^84^ According to the study by P. I. Milner, the pathological mechanism of Keshin‐Beck disease is that free radicals induce chondrocyte apoptosis through cytokines associated with cartilage and participate in the regulation of chondrocyte differentiation.^68^, ^97^ The expression levels of FGF‐8 and FGFR3 are significantly increased in the articular cartilage of patients with Kashin‐Beck disease. In cell‐based experiments, hypertrophic chondrocytes treated with the free radical donor 3‐morpholinosydnonimine (SIN‐1) exhibited significantly increased levels of the FGF‐8 and FGFR3 mRNAs and proteins. Thus, hypertrophic chondrocytes upregulate the FGF‐8 and FGFR3 under oxidative conditions, resulting in abnormal chondrocyte terminal differentiation and degradation of chondrocyte extracellular matrix and inducing the formation of abnormal bone segments.^78^, ^92^, ^98^ Considering that oxidative stress leads to abnormal expression of differentiation factors FGF‐8 and FGFR3, which leads to chondrogenic differentiation, FGF‐8 and FGFR3 may be potential targets for the treatment of Keshin‐Beck disease, but further experimental and clinical studies are needed.

### 3.3 FGF‐18 and osteoarthritis

Considering the structural homology of FGF‐18 and FGF‐8, understanding the role of FGF‐18 in cartilage diseases may provide some instructions for us to better understand FGF‐8. FGF‐18 in osteoarthritis is different from FGF‐8 because it mainly protects cartilage.^2^, ^90^ FGF‐18, a high‐affinity ligand for FGFR3, is the only FGF‐based drug currently used in clinical trials for osteoarthritis.^4^, ^8^, ^99^ FGF‐18 can significantly enhance anabolism during articular cartilage repair by activating the MEK‐ERK pathway to induce chondrocyte proliferation.^14^, ^81^ FGF‐18 binds to FGFR3 and inhibits cartilage hypertrophy‐related factors and precursor inflammatory cytokines. Moreover, it inhibits the formation of MMPs, inhibits the release and consumption of glucosaminoglycans in cartilage and promotes the synthesis of extracellular matrix by upregulating TIMP‐1. Eventually, FGF‐18 can stimulate the type Ⅱ collagen's production, proteoglycan accumulation and chondrocyte proliferation.^100^, ^101^, ^102^ Therefore, FGF‐18 may be considered as a molecule that can protect against articular cartilage degeneration. The difference between FGF‐18 and FGF‐8 in the progression of cartilage disease suggests that the FGF‐8 family has a dual role in the progression of cartilage disease, which may contribute to our complete understanding of the molecular mechanism of FGF‐8 in cartilage diseases.

## 4 CONCLUSIONS AND PERSPECTIVES

Cartilage tissue supports normal body movement and bone formation so the development of cartilage tissue is important for human growth and development. And it has been proved that FGF‐8 signalling pathway is considered a vital pathway to regulate early physiological activities in the cartilage formation, including but not limited to promoting migration of mesenchymal stem cells, differentiation into chondrocytes and proliferation of chondrocytes. Considering FGF‐8 remains low level at the normal cartilage, and the normal cartilage tissue is manifested in the interaction in the dynamic balance of catabolism and anabolism, the role that FGF‐8 plays in chondrogenesis and cartilage stabilization is different. In different stages of the growth and development of limbs and joints, different types of FGFRs (especially FGFR3 and FGFR1) successively bind to FGF‐8 to activate downstream signalling pathways such as MAPK‐MEK‐ERK, PI3K‐AKT and BMP with different intensities. The complex biological effects of all signalling pathways are cumulative and manifest as the ultimate result of limb and joint development. At the same time, FGF‐8 and FGFR are maintained in long‐term dynamic balance in normal mature cartilage tissue to ensure the normal motor function of the joints and limbs of the body.

In the osteoarthritis model, FGF‐8 mainly binds to FGFR1 and activates downstream MAPK‐ERK signalling, resulting in the production of MMP‐3 and PGE2, the degradation of type II collagen fibres in the extracellular matrix and a decrease in the aggrecan content. Levels of endogenous inhibitors, such as tissue inhibitor of metalloproteinases 3 (TIMP‐3) and TIMP‐1, are also reduced, resulting in damage to chondrocytes and chondro‐environments. At the same time, the decomposed extracellular matrix is released into synovial fluid of the joint, aggravating cartilage tissue destruction. Eventually, cartilage homeostasis shifts towards cartilage degradation. When FGF‐8 is applied in combination with interleukin‐1, the degradation of cartilage matrix in the inflammatory joints is more active. The use of anti‐FGF‐8 antibodies significantly inhibits cartilage destruction and extracellular matrix decomposition. Besides, according to the study by P. I. Milner, the pathological mechanism of Kashin‐Beck disease, another type of osteoarthritis, is that free radicals induce chondrocyte apoptosis through cytokines associated with cartilage and participate in the regulation of oxidative chondrogenic differentiation. The expression of FGF‐8 and FGFR3 is significantly increased in the articular cartilage of patients with Kashin‐Beck disease. Therefore, FGF‐8 may be a potential therapeutic target for patients with osteoarthritis.

At present, the research on FGF‐8 and chondrocytes mainly focuses on the changes of downstream signal pathways in chondrocytes stimulated by FGF‐8. However, the relationship between the changes of these complex pathways has not been clear. After chondrocytes are stimulated by FGF‐8, it is necessary to design and carry out cell experiments through the action of specific pathway inhibitors to explore the pathways involved in response and regulation in chondrocytes after FGF‐8 stimulation. At the same time, considering that the dynamic balance of cartilage tissue is the result of the joint action of four layers of cells in cartilage, adding or deleting FGF‐8 in the four cells to judge the role of FGF‐8 in different stages of cartilage development may become a direction to explore the mechanism of FGF‐8 promoting cartilage dynamic balance in normal cartilage tissue. In future animal experiments, designing knockdown of FGF‐8 in articular cartilage of mice at different ages may also help to verify the role of FGF‐8 in cartilage tissue.

Besides, in osteoarthritis, cartilage is characterized by destruction of cartilage matrix and lysis of chondrocytes. Existing studies have shown that the progress of OA is mainly the disorder of chondrocyte metabolism. Therefore, if we want to determine the specific role of FGF‐8 in osteoarthritis chondrocytes, it may be a feasible scheme to detect related proteins from the metabolism of pathological chondrocytes such as energy metabolism, glucose metabolism and lipid metabolism in future. Considering that FGF‐8 can induce chondrogenesis of mesenchymal cells. And FGF‐18, a homologous family factor of FGF‐8, has been proved to have a restorative effect in OA. The repair effect of FGF‐8 on cartilage can be speculated to a certain extent. In future, stem cell tissue engineering induced by FGF‐8 will have a very broad prospect for the repair of damaged cartilage.

In conclusion, we have reason to believe that FGF‐8 plays an important role in cartilage and can be regarded as a target for the treatment of osteoarthritis in future. However, a large number of experiments are still needed to explore its role in different stages of chondrocytes and pathological cartilage, as well as its application in the repair of damaged cartilage in future.

## CONFLICT OF INTEREST

The authors declare that no competing interests exist.

## AUTHOR CONTRIBUTIONS

**Haoran Chen:** Data curation (lead); Writing – original draft (lead); Writing – review & editing (equal). **Yujia Cui:** Conceptualization (supporting); Data curation (supporting); Formal analysis (supporting); Investigation (supporting); Methodology (supporting); Resources (supporting); Validation (supporting); Writing – review & editing (supporting). **Demao Zhang:** Investigation (supporting); Methodology (supporting); Resources (supporting); Software (supporting); Supervision (supporting); Validation (supporting); Visualization (supporting). **jing xie:** Conceptualization (lead); Methodology (lead); Project administration (lead); Resources (lead); Software (lead); Supervision (lead); Writing – original draft (lead); Writing – review & editing (lead). **XUE ZHOU:** Conceptualization (lead); Data curation (lead); Funding acquisition (lead); Project administration (lead); Supervision (lead); Validation (lead); Visualization (lead); Writing – original draft (lead); Writing – review & editing (lead).

## Data Availability

Any data involving this study are available from the corresponding author on request.
